# Editorial: Macrophage Plasticity in Sterile and Pathogen-Induced Inflammation

**DOI:** 10.3389/fimmu.2021.823023

**Published:** 2021-12-17

**Authors:** Ioannis Kourtzelis, James Hewitson, Thierry Roger

**Affiliations:** ^1^ Hull York Medical School, York Biomedical Research Institute, University of York, York, United Kingdom; ^2^ Department of Biology, York Biomedical Research Institute, University of York, York, United Kingdom; ^3^ Infectious Diseases Service, Department of Medicine, Lausanne University Hospital and University of Lausanne, Epalinges, Switzerland

**Keywords:** macrophage plasticity, resolution of inflammation, reprograming, memory, sterile inflammation

Macrophages are dynamic and heterogenous group of immune cells that play multiple instrumental roles in homeostasis, surveillance and host defence. Owing to their remarkable functional plasticity, macrophages are involved in the initiation, amplification, and termination of immune responses. The localization of macrophages in a variety of tissues and their capacity to respond in a timely manner to a plethora of stimuli such as pathogen-associated molecular patterns (PAMPs) and danger-associated molecular patterns (DAMPs) underscores their importance in the modulation of inflammatory responses. Distinct pathogens induce characteristic and divergent profiles of macrophage activation that can either boost or dampen inflammation. Besides PAMPs, DAMPs released by injured or stressed cells during sterile inflammatory processes contribute to the shaping of macrophage phenotype. Macrophages are major determinants in disease models that are linked to sterile inflammation. Pathogenesis of sterile inflammatory diseases including metabolic diseases is affected by macrophage activity. In all these instances, macrophages orchestrate immune responses through close interactions with both immune and non-immune cells.

Our aim with this Frontiers in Immunology Research Topic *“Macrophage Plasticity in Sterile and Pathogen-Induced Inflammation”* is to showcase some of the latest mechanistic insights into how macrophages control responses to pathogens and sterile inflammatory insults. We herein give an overview of this Frontiers in Immunology topic which includes 9 original articles and 8 (mini) review articles.

Pantazi et al. describe a novel mechanism by which activation of angiotensin converting enzyme 2 (ACE2) may form the basis of an anti-inflammatory therapy that blocks the cytokine storm observed in COVID-19 patients. They propose that treatment of macrophages with the SARS-CoV-2 spike (S) protein results in the hyper-responsiveness of macrophages to Toll-like receptor (TLR) signals thereby promoting expression of pro-inflammatory mediators. The authors present evidence that treatment with S protein suppresses the inactive kinase isoform of IL-1R-associated kinase (IRAK)-M that is a central regulator of TLR responsiveness. They conclude that activation of ACE2 may represent a therapeutic strategy to interfere with the development of cytokine storm observed in COVID-19 patients.

Knoll et al. review the key concepts and findings on the role of peripheral blood monocytes and pulmonary macrophages in COVID-19. Blood monocytes in patients with moderate COVID-19 have an inflammatory phenotype linked to interferon-stimulated gene expression and cellular dysfunction. Along the same line, monocytes-derived pulmonary macrophages from COVID-19 patients exhibit a hyperactivated state that promotes pro-inflammatory cytokine release and subsequent tissue damage at the site of infection.

Li et al. also demonstrate the role of macrophages in lung inflammation. In particular, the authors show that expression of total and de-oligomerized surfactant protein D (SP-D) is elevated following the lipopolysaccharide (LPS)-induced acute lung injury (ALI). Consistently, treatment with de-oligomerized SP-D resulted in enhanced acute lung injury linked to classically activated/M1 macrophages and pro-inflammatory cytokine production. The authors propose targeting de-oligomerized SP-D as a strategy to treatment of ALI and acute respiratory distress syndrome (ARDS).

Long non-coding RNAs (lncRNAs) are endogenous factors that have been linked to the regulation of innate inflammatory responses including the control of macrophage polarization. Ahmad et al. elucidate a novel role for the lncRNAs RN7SK and GAS5 in monocyte to macrophage differentiation into M1 (using granulocyte-macrophage colony-stimulating factor) and M2 (using macrophage colony-stimulating factor) macrophages. The knockdown of either RN7SK or GAS5 during the differentiation process resulted in the downregulation of M2 surface markers and concomitant increase in M1 markers, alongside enhanced antigen uptake and processing. In addition, the absence of RN7SK promoted the phagocytosis of *Escherichia coli* by M1 and M2 macrophages. This study reinforces the concept of lncRNA-mediated epigenetic regulation of macrophage differentiation and innate immune functions.

Macrophage activity has been implicated in the regulation of inflammatory responses during arthritis. Abji et al. used single cell RNA sequencing to identify cell populations in the synovial fluid from patients with psoriatic arthritis compared to those with osteoarthritis and rheumatoid arthritis. They report macrophages are one of the main cellular components in the synovial fluid of patients with psoriatic arthritis. As multiple proteinases are present in arthritic joint, the authors characterised the proteinase activity in synovial fluid samples. Trypsin-like serine proteinase activity was particularly elevated in psoriatic arthritis. Tryptase-6 was identified as a possible activator of proteinase-activated receptor 2 (PAR2) signaling that impacts on the production of monocyte chemoattractant protein-1. Thus, tryptase-6/PAR2 axis might be targeted to inhibit monocytes/macrophages recruitment in psoriatic arthritis joints.

Macrophage-dependent responses have also been linked to the orchestration of bone remodelling and the maintenance of normal bone morphology. Yao et al. review our current understanding of the contribution of monocytes and tissue macrophages to osteoclast formation. The authors further describe the role of macrophages and osteoclasts on bone damage mediated by inflammation and immune diseases.

Ross et al. review the role of macrophages in the onset and resolution rheumatoid arthritis and allergic asthma in terms of “The Good, the Bad, and the Gluttony”. The authors contrast macrophage diversity and regulatory function in healthy individuals with joint macrophages from rheumatoid arthritis and lung macrophages in allergic asthma. They emphasize the role of efferocytosis, and extracellular vesicles derived from apoptotic cells on the control of macrophage phenotype during inflammation resolution and in the control of adaptive immune responses.

Inflammasome activity can modulate the function of monocytes, precursors of macrophages. EMR2/ADGRE2, the human paralogue of the mouse F4/80 macrophage antigen, is an adhesion G protein-coupled receptor expressed by myeloid cells. I et al. provide evidence that EMR2/ADGRE2 signalling mediates the activation of the NOD-like receptor family, pyrin domain containing 3 (NLRP3) inflammasome in human monocytes. Specifically, the authors demonstrate that stimulation of EMR2/ADGRE2 using an agonistic monoclonal antibody activates the phospholipase C-β pathway, inducing downstream signalling through the Akt, mitogen-activated protein kinase (MAPK) and nuclear factor-κB (NF-κB) pathways. This results in calcium mobilization and efflux of potassium ions leading to the activation of the NLRP3 inflammasome. Thus, EMR2/ADGRE2 is a novel GPCRs that can activate the NLRP3 inflammasome in human myeloid cells.

Regulation of death in immune cells, including macrophages, is of paramount importance in shaping the outcome of inflammatory responses. Fischer et al. review recent findings on the regulation of the proinflammatory form of cell death called pyroptosis, mediated by membrane pore-forming proteins called gasdermins. The assembled gasdermin D pores measure up to 20 nm in diameter and allow the secretion of small molecules including mature IL-1β generated through active inflammasome. The authors further describe endogenous mechanisms that could be targeted to block inflammation through modulation of pyroptosis. They conclude gasdermins are promising targets for therapeutic targeting to modulate cell death and immune responses.

A recent breakthrough in immunology has been the demonstration in mammals that the innate immune system is able to recall a first challenge to respond more efficiently to a secondary challenge. This phenomenon has been called trained immunity. Macrophage-dependent responses have been associated with the induction and orchestration of innate immune memory. Jentho and Weis summarize experimental data supporting the involvement of host-derived DAMPs on the modulation of innate immune memory. They end their mini review by highlighting several open questions that will drive the field of trained immunity the next few years.

Macrophages have substantial roles at multiple points during the process of reproduction. They influence the cycling uterus and gestational processes including implantation, placentation, and parturition. Chambers et al. discuss the impact of macrophage polarization in the development of adverse obstetric outcomes. The authors propose that the characterisation of environmental influences may unravel new mechanisms regulating macrophage polarization at the maternal-fetal interface, thus supporting the development of approaches to alleviate adverse outcomes.

Metabolic alterations have been described as key determinants in macrophage inflammatory responses. Whilst classically activated or M1 macrophages switch from oxidative phosphorylation to glycolysis and pentose phosphate pathway to increase antimicrobial functions, alternatively activated M2 macrophages maintain oxidative metabolism through fatty acid oxidation, providing energy for a sustained tissue repair. Verberk et al. provide evidence that absence of the metabolic enzyme ATP citrate lyase (Acly) in macrophages leads to hyperinflammatory gene expression in response to LPS stimulation *in vitro.* Despite this, myeloid-specific deletion of Acly did not worsen inflammation in *in vivo* models of endotoxin-induced peritonitis, obesity-induced low-grade inflammation or experimental autoimmune encephalomyelitis. As such, therapeutic targeting of Acly with macrophage-specific Acly inhibitors may not drive beneficial effects in all inflammatory disorders.

Maretti-Mira et al. describe a novel mechanism of macrophage activation mediated by the different degrees of cholesterol oxidation. Using human peripheral blood CD14^+^ monocyte-derived macrophages and hepatic macrophages (i.e. Kupffer cells), the authors show that highly oxidized low-density lipoproteins (HoxLDL) affect global gene expression in M2-like macrophages and instead promote M4-like polarization. This involves decreased expression *IL10*, *MRC1*, and *CD163* and reduced phagocytic capacity. HoxLDL-derived M4 polarization promotes *in vitro* neutrophil recruitment and generation of neutrophil extracellular traps (NETs). These findings provided a link between oxidized LDL and alteration of the functions of blood-derived and liver macrophages.

Macrophage phagocytic activity is a major contributor to the clearance of red blood cells and hemoproteins both during hemolytic disorders and after tissue injury. Pfefferlé et al. utilise a model of anti-CD40 macrophage-driven sterile liver inflammation to demonstrate phenylhydrazine-mediated acute erythrophagocytosis blocks macrophage activation, inflammatory cytokine release syndrome and necrotizing hepatitis. Moreover, treatment of liver macrophages with heme-albumin complexes results in an anti-inflammatory phenotype that protects against sterile liver inflammation. Together, this work suggests heme signalling suppresses proinflammatory macrophage function and protects from sterile liver inflammation.

Tissue-resident and recruited macrophages play a central part in the development of inflammation and fibrosis progression in the liver. Zwicker et al. review our current understanding of macrophage heterogeneity and plasticity in the liver, and the responses of hepatic macrophages to both pathogen-induced and sterile inflammation. Similarly, Singanayagam and Triantafyllou summarize recent findings related to the phenotypic and functional alterations that macrophages undergo during liver failures. The authors highlight potential therapeutic options for targeting macrophages in liver diseases.

Interactions between macrophages and other types of immune or non-immune cells modulate inflammatory responses. In this respect, Bhattacharjee et al. focus on the crosstalk between epithelial cells and dermal macrophages. Using a model of integrin-β1 (Itgβ1) knockout skin, they present data indicating Itgβ1-/- skin epithelial cells produce cytokines, chemokines and DAMPs that activate and enhance an M2-like pro-remodelling phenotype of tissue resident and monocyte-derived macrophages. These data highlight the role of the epithelial-immune crosstalk in priming macrophage cell fate.

In summary, this Research Topic provides better understanding of the mechanistic role of macrophages in multiple different inflammatory conditions. Many important questions remain unsolved and given their pivotal function in host defence and the potential danger posed by uncontrolled inflammation, understanding macrophage activity is essential for harnessing pathological mechanisms. Identification of novel mechanisms that regulate plasticity and immune functions of macrophages will unravel new therapeutic strategies.

## Author Contributions

IK, JH, and TR have contributed to the writing of this editorial article. All authors contributed to the article and approved the submitted version.

## Funding

IK acknowledges support from grant from the Royal Society (RGS\R2\202032). JH was supported by the Academy of Medical Science and Global Challenges Research Fund (SBF003\1096). TR was supported by the Swiss National Science Foundation (173123), the Carigest Foundation (Switzerland), and the European Union Horizon 2020 Marie Skłodowska-Curie Action Innovative Training Network European Sepsis Academy (676129), and Horizon 2020 grants ImmunoSep (847422) and HDM-FUN (847507).

## Conflict of Interest

The authors declare that the research was conducted in the absence of any commercial or financial relationships that could be construed as a potential conflict of interest.

## Publisher’s Note

All claims expressed in this article are solely those of the authors and do not necessarily represent those of their affiliated organizations, or those of the publisher, the editors and the reviewers. Any product that may be evaluated in this article, or claim that may be made by its manufacturer, is not guaranteed or endorsed by the publisher.

